# 1-(2-Eth­oxy-2-methyl-2*H*-chromen-3-yl)ethanone

**DOI:** 10.1107/S1600536809008502

**Published:** 2009-03-14

**Authors:** Afsaneh Zonouzi, Mojtaba Biniaz, Hossein Rahmani, Seik Weng Ng

**Affiliations:** a Department of Chemistry, College of Science, University of Tehran, PO Box 13145-143, Tehran, Iran; b Institute of Chemical Industries, Iranian Research Organization for Science and Technology, PO Box 15815-358, Tehran, Iran; c Department of Chemistry, University of Malaya, 50603 Kuala Lumpur, Malaysia

## Abstract

The C*sp* ^3^ atom of the chromenyl fused-ring system in the title compound, C_14_H_16_O_3_, deviates by 0.407 (2) Å from the plane of the other atoms (r.m.s. deviation = 0.041 Å). The eth­oxy substituent occupies a pseudo-axial position.

## Related literature

For the synthesis, see: Zonouzi *et al.* (2008*b* ▶). For related crystal structures, see: Bardajee *et al.* (2007 ▶); Zhan & Lin (2006 ▶); Zonouzi *et al.* (2008*a* ▶,*b* ▶).
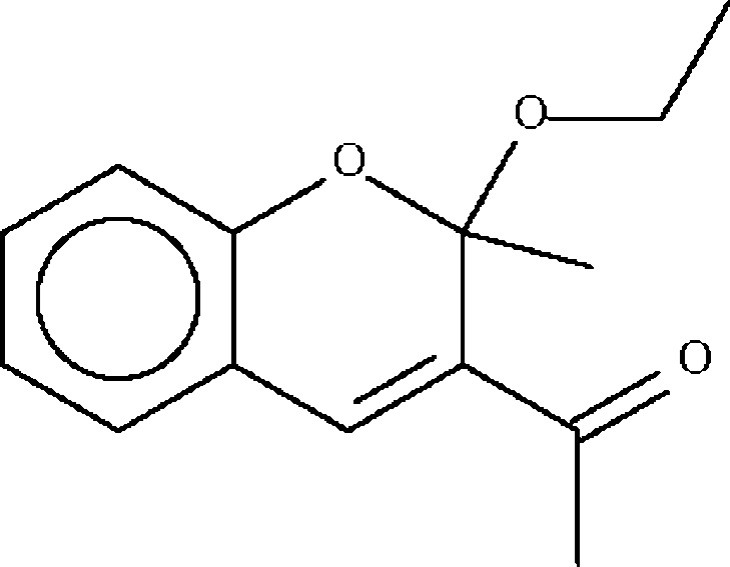


## Experimental

### 

#### Crystal data

C_14_H_16_O_3_
*M* *_r_* = 232.27
Monoclinic, 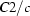

*a* = 20.0084 (5) Å
*b* = 7.2637 (2) Å
*c* = 19.1056 (5) Å
β = 123.294 (1)°
*V* = 2320.96 (11) Å^3^
*Z* = 8
Mo *K*α radiation
μ = 0.09 mm^−1^
*T* = 123 K
0.25 × 0.20 × 0.15 mm

#### Data collection

Bruker SMART APEX diffractometer
Absorption correction: none
7793 measured reflections
2655 independent reflections
2123 reflections with *I* > 2σ(*I*)
*R*_int_ = 0.035

#### Refinement

*R*[*F* ^2^ > 2σ(*F* ^2^)] = 0.047
*wR*(*F* ^2^) = 0.140
*S* = 1.03
2655 reflections
157 parameters
H-atom parameters constrained
Δρ_max_ = 0.36 e Å^−3^
Δρ_min_ = −0.43 e Å^−3^

### 

Data collection: *APEX2* (Bruker, 2008 ▶); cell refinement: *SAINT* (Bruker, 2008 ▶); data reduction: *SAINT*; program(s) used to solve structure: *SHELXS97* (Sheldrick, 2008 ▶); program(s) used to refine structure: *SHELXL97* (Sheldrick, 2008 ▶); molecular graphics: *X-SEED* (Barbour, 2001 ▶); software used to prepare material for publication: *publCIF* (Westrip, 2009 ▶).

## Supplementary Material

Crystal structure: contains datablocks global, I. DOI: 10.1107/S1600536809008502/tk2390sup1.cif

Structure factors: contains datablocks I. DOI: 10.1107/S1600536809008502/tk2390Isup2.hkl

Additional supplementary materials:  crystallographic information; 3D view; checkCIF report

## Figures and Tables

**Fig. 1.**
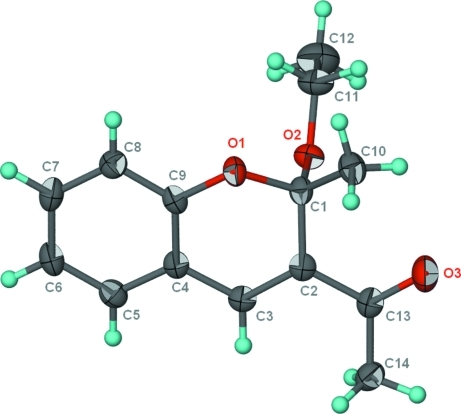
Thermal ellisoid plot (Barbour, 2001) of C14H16O3; probability levels are set at 70% and H-atoms are drawn as spheres of arbitrary radius.

## supplementary crystallographic information

### Experimental

The compound was synthesized by using a reported method (Zonouzi *et al.*, 2008*b*). Crystals were obtained by recrystallization from ethanol.

### Refinement

Carbon-bound H-atoms were placed in calculated positions (C–H 0.95 to 0.99 Å) and were included in the refinement in the riding model approximation, with *U*(H) set to 1.2 to 1.5U_eq_(C).

### Crystal data

**Table:**

C_14_H_16_O_3_	*F*(000) = 992
*M**_r_* = 232.27	*D*_x_ = 1.329 Mg m^−^^3^
Monoclinic, *C*2/*c*	Mo *K*α radiation, λ = 0.71073 Å
Hall symbol: -C 2yc	Cell parameters from 2233 reflections
*a* = 20.0084 (5) Å	θ = 2.4–28.1°
*b* = 7.2637 (2) Å	µ = 0.09 mm^−^^1^
*c* = 19.1056 (5) Å	*T* = 123 K
β = 123.294 (1)°	Irregular block, colorless
*V* = 2320.96 (11) Å^3^	0.25 × 0.20 × 0.15 mm
*Z* = 8	

### Data collection

**Table:**

Bruker SMART APEX diffractometer	2123 reflections with *I* > 2σ(*I*)
Radiation source: fine-focus sealed tube	*R*_int_ = 0.035
graphite	θ_max_ = 27.5°, θ_min_ = 2.4°
ω scans	*h* = −21→25
7793 measured reflections	*k* = −9→9
2655 independent reflections	*l* = −24→23

### Refinement

**Table:**

Refinement on *F*^2^	Primary atom site location: structure-invariant direct methods
Least-squares matrix: full	Secondary atom site location: difference Fourier map
*R*[*F*^2^ > 2σ(*F*^2^)] = 0.047	Hydrogen site location: inferred from neighbouring sites
*wR*(*F*^2^) = 0.140	H-atom parameters constrained
*S* = 1.03	*w* = 1/[σ^2^(*F*_o_^2^) + (0.0739*P*)^2^ + 1.4605*P*] where *P* = (*F*_o_^2^ + 2*F*_c_^2^)/3
2655 reflections	(Δ/σ)_max_ < 0.001
157 parameters	Δρ_max_ = 0.36 e Å^−^^3^
0 restraints	Δρ_min_ = −0.43 e Å^−^^3^

### Fractional atomic coordinates and isotropic or equivalent isotropic displacement parameters (Å^2^)

**Table:**

	*x*	*y*	*z*	*U*_iso_*/*U*_eq_	
O1	0.13572 (6)	0.55707 (15)	0.32959 (6)	0.0208 (3)	
O2	0.17621 (6)	0.25748 (14)	0.32719 (6)	0.0211 (3)	
O3	0.36134 (7)	0.35871 (19)	0.38669 (7)	0.0350 (3)	
C1	0.19962 (9)	0.4438 (2)	0.33800 (9)	0.0193 (3)	
C2	0.27375 (9)	0.4496 (2)	0.42713 (9)	0.0189 (3)	
C3	0.26440 (9)	0.4851 (2)	0.49036 (9)	0.0198 (3)	
H3	0.3098	0.4792	0.5463	0.024*	
C4	0.18788 (9)	0.5318 (2)	0.47624 (9)	0.0190 (3)	
C5	0.17449 (9)	0.5519 (2)	0.54044 (9)	0.0223 (3)	
H5	0.2169	0.5303	0.5969	0.027*	
C6	0.10015 (10)	0.6026 (2)	0.52253 (10)	0.0244 (3)	
H6	0.0917	0.6164	0.5666	0.029*	
C7	0.03757 (10)	0.6336 (2)	0.43999 (10)	0.0239 (3)	
H7	−0.0135	0.6686	0.4280	0.029*	
C8	0.04917 (9)	0.6137 (2)	0.37524 (9)	0.0222 (3)	
H8	0.0063	0.6340	0.3189	0.027*	
C9	0.12413 (9)	0.5638 (2)	0.39363 (9)	0.0185 (3)	
C10	0.20936 (10)	0.5212 (2)	0.27015 (9)	0.0250 (4)	
H10A	0.1566	0.5384	0.2185	0.037*	
H10B	0.2407	0.4352	0.2597	0.037*	
H10C	0.2371	0.6399	0.2885	0.037*	
C11	0.11063 (10)	0.2021 (2)	0.24546 (9)	0.0282 (4)	
H11A	0.1250	0.2167	0.2037	0.034*	
H11B	0.0628	0.2781	0.2278	0.034*	
C12	0.09445 (12)	0.0053 (3)	0.25244 (12)	0.0409 (5)	
H12A	0.0504	−0.0390	0.1978	0.061*	
H12B	0.0798	−0.0068	0.2935	0.061*	
H12C	0.1425	−0.0678	0.2706	0.061*	
C13	0.35266 (9)	0.3964 (2)	0.44326 (9)	0.0218 (3)	
C14	0.42348 (9)	0.3868 (2)	0.53284 (9)	0.0256 (4)	
H14A	0.4721	0.3623	0.5337	0.038*	
H14B	0.4152	0.2876	0.5621	0.038*	
H14C	0.4291	0.5043	0.5608	0.038*	

### Atomic displacement parameters (Å^2^)

**Table:**

	*U*^11^	*U*^22^	*U*^33^	*U*^12^	*U*^13^	*U*^23^
O1	0.0232 (5)	0.0224 (6)	0.0196 (5)	0.0066 (4)	0.0136 (4)	0.0041 (4)
O2	0.0220 (6)	0.0178 (6)	0.0183 (5)	0.0008 (4)	0.0077 (4)	0.0006 (4)
O3	0.0311 (7)	0.0482 (8)	0.0313 (6)	0.0106 (6)	0.0207 (5)	0.0023 (5)
C1	0.0206 (7)	0.0186 (7)	0.0207 (7)	0.0030 (6)	0.0126 (6)	0.0013 (5)
C2	0.0208 (7)	0.0146 (7)	0.0216 (7)	0.0000 (6)	0.0119 (6)	0.0015 (5)
C3	0.0203 (7)	0.0171 (7)	0.0202 (7)	−0.0003 (6)	0.0101 (6)	0.0003 (5)
C4	0.0222 (7)	0.0155 (7)	0.0212 (7)	−0.0011 (6)	0.0131 (6)	−0.0001 (5)
C5	0.0258 (8)	0.0206 (8)	0.0211 (7)	0.0000 (6)	0.0132 (6)	0.0001 (6)
C6	0.0323 (9)	0.0222 (8)	0.0270 (7)	0.0007 (7)	0.0215 (7)	−0.0003 (6)
C7	0.0238 (8)	0.0213 (8)	0.0321 (8)	0.0023 (6)	0.0189 (7)	0.0005 (6)
C8	0.0224 (8)	0.0199 (7)	0.0236 (7)	0.0030 (6)	0.0122 (6)	0.0017 (6)
C9	0.0237 (7)	0.0142 (7)	0.0200 (7)	0.0002 (6)	0.0135 (6)	0.0006 (5)
C10	0.0318 (9)	0.0237 (8)	0.0238 (7)	0.0049 (7)	0.0180 (7)	0.0043 (6)
C11	0.0257 (8)	0.0265 (9)	0.0207 (7)	−0.0003 (7)	0.0054 (6)	−0.0016 (6)
C12	0.0394 (11)	0.0386 (11)	0.0360 (9)	−0.0071 (9)	0.0151 (8)	−0.0031 (8)
C13	0.0229 (8)	0.0176 (7)	0.0264 (7)	0.0003 (6)	0.0145 (6)	0.0007 (6)
C14	0.0201 (7)	0.0249 (8)	0.0299 (8)	−0.0002 (6)	0.0125 (6)	−0.0008 (6)

### Geometric parameters (Å, °)

**Table:**

O1—C9	1.3654 (17)	C7—C8	1.386 (2)
O1—C1	1.4531 (18)	C7—H7	0.9500
O2—C1	1.4101 (18)	C8—C9	1.388 (2)
O2—C11	1.4397 (17)	C8—H8	0.9500
O3—C13	1.2164 (18)	C10—H10A	0.9800
C1—C10	1.520 (2)	C10—H10B	0.9800
C1—C2	1.5259 (19)	C10—H10C	0.9800
C2—C3	1.345 (2)	C11—C12	1.488 (3)
C2—C13	1.484 (2)	C11—H11A	0.9900
C3—C4	1.442 (2)	C11—H11B	0.9900
C3—H3	0.9500	C12—H12A	0.9800
C4—C5	1.400 (2)	C12—H12B	0.9800
C4—C9	1.4006 (19)	C12—H12C	0.9800
C5—C6	1.381 (2)	C13—C14	1.510 (2)
C5—H5	0.9500	C14—H14A	0.9800
C6—C7	1.392 (2)	C14—H14B	0.9800
C6—H6	0.9500	C14—H14C	0.9800
			
C9—O1—C1	119.55 (11)	O1—C9—C4	120.41 (13)
C1—O2—C11	117.31 (11)	C8—C9—C4	121.18 (13)
O2—C1—O1	109.01 (12)	C1—C10—H10A	109.5
O2—C1—C10	114.69 (12)	C1—C10—H10B	109.5
O1—C1—C10	102.32 (11)	H10A—C10—H10B	109.5
O2—C1—C2	103.36 (11)	C1—C10—H10C	109.5
O1—C1—C2	111.41 (11)	H10A—C10—H10C	109.5
C10—C1—C2	116.12 (13)	H10B—C10—H10C	109.5
C3—C2—C13	121.33 (13)	O2—C11—C12	106.55 (13)
C3—C2—C1	118.58 (13)	O2—C11—H11A	110.4
C13—C2—C1	119.74 (13)	C12—C11—H11A	110.4
C2—C3—C4	122.28 (13)	O2—C11—H11B	110.4
C2—C3—H3	118.9	C12—C11—H11B	110.4
C4—C3—H3	118.9	H11A—C11—H11B	108.6
C5—C4—C9	118.45 (14)	C11—C12—H12A	109.5
C5—C4—C3	123.72 (13)	C11—C12—H12B	109.5
C9—C4—C3	117.81 (13)	H12A—C12—H12B	109.5
C6—C5—C4	120.60 (14)	C11—C12—H12C	109.5
C6—C5—H5	119.7	H12A—C12—H12C	109.5
C4—C5—H5	119.7	H12B—C12—H12C	109.5
C5—C6—C7	120.05 (14)	O3—C13—C2	121.91 (13)
C5—C6—H6	120.0	O3—C13—C14	119.59 (14)
C7—C6—H6	120.0	C2—C13—C14	118.49 (13)
C8—C7—C6	120.47 (14)	C13—C14—H14A	109.5
C8—C7—H7	119.8	C13—C14—H14B	109.5
C6—C7—H7	119.8	H14A—C14—H14B	109.5
C7—C8—C9	119.25 (14)	C13—C14—H14C	109.5
C7—C8—H8	120.4	H14A—C14—H14C	109.5
C9—C8—H8	120.4	H14B—C14—H14C	109.5
O1—C9—C8	118.29 (13)		
			
C11—O2—C1—O1	66.57 (15)	C3—C4—C5—C6	177.92 (15)
C11—O2—C1—C10	−47.43 (18)	C4—C5—C6—C7	0.3 (2)
C11—O2—C1—C2	−174.83 (12)	C5—C6—C7—C8	0.1 (2)
C9—O1—C1—O2	75.85 (15)	C6—C7—C8—C9	−0.5 (2)
C9—O1—C1—C10	−162.31 (12)	C1—O1—C9—C8	−157.01 (13)
C9—O1—C1—C2	−37.58 (17)	C1—O1—C9—C4	26.77 (19)
O2—C1—C2—C3	−90.42 (15)	C7—C8—C9—O1	−175.65 (13)
O1—C1—C2—C3	26.51 (19)	C7—C8—C9—C4	0.5 (2)
C10—C1—C2—C3	143.09 (15)	C5—C4—C9—O1	175.92 (13)
O2—C1—C2—C13	82.91 (16)	C3—C4—C9—O1	−2.3 (2)
O1—C1—C2—C13	−160.17 (13)	C5—C4—C9—C8	−0.2 (2)
C10—C1—C2—C13	−43.59 (19)	C3—C4—C9—C8	−178.45 (14)
C13—C2—C3—C4	−178.17 (14)	C1—O2—C11—C12	−176.06 (14)
C1—C2—C3—C4	−5.0 (2)	C3—C2—C13—O3	177.78 (15)
C2—C3—C4—C5	173.50 (14)	C1—C2—C13—O3	4.6 (2)
C2—C3—C4—C9	−8.3 (2)	C3—C2—C13—C14	−1.4 (2)
C9—C4—C5—C6	−0.2 (2)	C1—C2—C13—C14	−174.50 (13)
